# Superior immune responses from thermostable, single-administration rabies vaccines prepared using atomic layer deposition

**DOI:** 10.1016/j.xphs.2025.103936

**Published:** 2025-08-06

**Authors:** Theodore Randolph, Holly Coleman, Amber M. Rauch, Hannah Hubert, Allysa E. Witeof, Kathryn D. Walker, Hans H. Funke, Robert L. Garcea

**Affiliations:** a Department of Chemical and Biological Engineering, University of Colorado Boulder, CO 80303, USA; b Department of Molecular, Cellular, and Developmental Biology and the BioFrontiers Institute, University of Colorado Boulder, CO 80303, USA

**Keywords:** Immunogenicity, Microparticles, Spray drying, Vaccines, Vaccine delivery, Atomic layer deposition

## Abstract

Shortcomings of current rabies virus (RABV) vaccines include thermal instability and the need for multiple injections to achieve protective immune responses. To overcome these drawbacks we describe a single-administration vaccine formulation prepared using a combination of spray-drying and atomic layer deposition (ALD) technologies. First, an inactivated RABV antigen is spray-dried from polysaccharide solutions to create glassy microparticles. ALD in a fluidized bed reactor is then used to deposit nanoscopic layers of alumina onto particle surfaces. The nanoscopic alumina coatings deposited by ALD provide a programmable delayed release of RABV antigen that enable both prime and boost vaccine functionality within a single injection. Immune responses to the resulting vaccines were tested following intramuscular administration in mice by measuring total anti-rabies IgG titers as well as neutralizing antibody titers. The immune responses to the spray-dried vaccines were unaffected by storage of the vaccines for 3 months at temperatures as high as 50 °C. ALD-coated rabies vaccine microparticles were also thermostable, with no changes in immunogenicity apparent after 3 months storage at 50 °C. Moreover, ALD-coated RABV vaccine microparticles induced IgG and neutralizing antibody titers nearly an order of magnitude higher than those generated in response to conventional liquid RABV vaccine formulations. Thus, spray-dried and ALD-coated RABV vaccine microparticle formulations offer both thermostability and superior immunogenicity from single vaccine administrations, with the potential for relaxed vaccine cold-chain requirements, antigen-sparing efficacy, and a reduced need for multiple administrations.

## Introduction

Infection by rabies virus (RABV) is estimated to result in approximately 60,000 worldwide human deaths annually^1^. RABV is endemic in many parts of Africa and Asia, where dog bites cause 99 % of cases^2^. In 2018, The World Health Organization, the World Organization for Animal Health and the Food and Agriculture Organization of the United Nations launched the Zero by 30 campaign, aimed to end endemic human deaths from subtropic rabies by 2030^3,4^. This multistep approach requires wide availability of RABV vaccines for pre-exposure prophylaxis (PrEP) and post-exposure prophylaxis (PEP), as well as supplies of RABV vaccine for mass immunization of dog populations. Unfortunately, the availability of RABV vaccines remains limited in developing areas.

Most rabies deaths are associated with a lack of available rabies PEP treatments, or delays in timely PEP administration^5^. A 5-dose schedule is currently recommended for PEP^6^, and failure to adhere to the dosing schedule may result in preventable mortality^7,8^. A 2022 study in New Delhi found that 45 % of animal bite patients who were advised to receive RABV PEP were non-compliant with the recommended 5-dose PEP schedule, with an additional fraction of the patients delaying treatments^9^.

Conventional rabies vaccine formulations require strict temperature control to maintain stability. For example, Rabivax-S, a recently developed lyophilized RABV vaccine^10^ requires storage at 2–8 °C and has a post-reconstitution refrigerated shelf life of only 6 h. Maintaining proper cold chain adds costs and complicates the logistics of vaccine delivery^11–15^. In addition, for RABV vaccines the logistics are further complicated by the need to administer multiple doses. Ideally, an optimal RABV vaccine should be sufficiently thermostable to allow storage and transportation outside the cold chain and be administered in as few doses as possible.

We recently developed a new approach for formulating vaccines that uses atomic layer deposition (ALD) techniques to apply nanoscopic, protective metal oxide layers to the surfaces of glassy microparticles formed by spray-drying. This process creates vaccine formulations that provide both thermostability and controlled release characteristics that allow multiple vaccine doses to be administered in single injections^16–18^.

Spray-drying can be used to embed vaccine antigens within glassy microparticles^19–21^. Briefly, the technique involves atomizing liquid formulations comprising vaccine components (*e.g*., antigens and adjuvants) and polysaccharides (*e.g*., hydroxyethyl starch and trehalose) to create mists of microdroplets which are then contacted with dry, heated gas streams. The microdroplets dry rapidly, causing the polysaccharides to form glasses that encapsulate the vaccines. At temperatures below the glass transition temperature, high viscosities within the glassy microparticles inhibit molecular motions required for many degradation reactions, leading to thermostabilization of the embedded vaccine components.

Control of spray-drying parameters yields spherical microparticles with tunable particle sizes compatible with the second technology: atomic layer deposition (ALD). ALD is a solvent-free technique that can be used to deposit nanometer-thick layers of metal oxides such as alumina on the surfaces of vaccine microparticles produced by spray-drying. In the case of ALD deposition of alumina, the process involves a two-part, self-limiting cycle wherein hydroxyl groups on particle surfaces are first allowed to react with trimethylaluminum vapor, followed by a second reaction in which the surfaces are exposed to water vapor. Each two-part cycle deposits a single molecular layer of alumina (Al_2_O_3_) approximately 2.3 Å thick on the particle surface and provides a regenerated layer of surface hydroxyl groups available for reaction in subsequent cycles, which may be used to deposit multiple layers of alumina. When the ALD reaction is conducted in fluidized bed reactors, mixing of the fluidizing gases as particles are being coated results in highly conformal, uniform coatings^17,22–24^. Tens to thousands of cycles may be employed, yielding alumina layers with precisely controlled, uniform thicknesses of 2–500 nm.

When applied to vaccine-containing particles, these nanoscopic ALD-deposited alumina layers serve multiple functions, including providing time-delayed booster doses of antigen^17,18^ and potentially serving as a vaccine adjuvant. *In vivo*, alumina coatings gradually erode, eventually releasing the vaccine contents of the inner microparticle in a pulsatile fashion. The time at which this release occurs depends linearly on the number of molecular layers of alumina applied; release is delayed by approximately one week for every 50 layers^18^. Importantly, especially for protein subunit vaccines which are sensitive to hydrolysis reactions, alumina coatings produced by ALD are impervious to water vapor^22^, affording protection against water-mediated degradation processes and blocking proteases from contacting proteins embedded within the ALD-coated microparticle core. Vaccine formulations providing release in multiple stages can be created by mixing particles that have been coated with different numbers of nanoscopic alumina layers.

Here, we first reformulated and spray-dried RABV antigen from Rabivax-S, a commercially available RABV vaccine. We then tested the thermal stability of the resulting dry powders following three months of storage at temperatures as high as 50 °C by measuring anti-RABV IgG responses and neutralizing antibody titers produced following administration of the vaccines in mice. No loss of immunogenicity was observed for the spray-dried powders, even after three months at the highest temperature storage conditions tested. We next used ALD to apply nanoscopic layers of alumina on the surfaces of the spray-dried RABV vaccine microparticles. Immunogenicity of the alumina-coated RABV vaccine was similarly unaffected by three months of storage at temperatures as high as 50 °C. Surprisingly, titers for both anti-RABV IgG and neutralizing antibodies induced by the alumina-coated vaccines in mice were nearly an order of magnitude higher than those generated by administration of a conventional liquid formulation of RABV antigen. Finally, we showed that the ALD coatings offered programmable delayed release of the killed rabies virus antigen, allowing formulation of vaccine doses that provided both prime and boost functionality within a single injection.

## Materials and methods

### Reagents

Endotoxin-free ammonium acetate was purchased from Avantor (Radnor, PA). Endotoxin-free trehalose dihydrate was from Pfanstiehl (Waukegan, IL). Hydroxyethyl Starch (HES) was from Fresenius Kabi (Bad Homburg, Germany). Endotoxin-free l-histidine was from Research Products International (Mount Prospect, IL). Sodium phosphate dibasic, potassium chloride, potassium phosphate monobasic, and sodium chloride were obtained from Sigma Aldrich (St. Louis, MO). Horseradish peroxidase-conjugated anti-mouse IgG (HRP) was from Promega (Madison, WI). Polysorbate 20 (Tween 20), polysorbate 80 (Tween 80), and Ultra TMB were from Thermo-Scientific (Waltham, MA). Cytiva HyClone Water for Injection (WFI), sulfuric acid, and Corning Costar^®^, 96 Well, Clear Flat Bottom Medium Binding Polystyrene Assay Plates were from Fischer Scientific (Waltham, MA). Powdered milk was obtained from Safeway. Bilicate glass vials (5 mL) with rubber stoppers were from Schott (Mainz, Germany). Nitrogen gas was from Airgas (Radnor, PA). Alhydrogel^®^ (aluminum hydroxide gel adjuvant) was obtained from Accurate Chemical & Scientific Corp (Westbury, NY). Methanol, HYDRANOL^™^ Composite 1, and formamide were obtained from Honeywell (Muskegon, MI). A human, anti-rabies monoclonal antibody (RAB-1 anti-rabies mAb), RabiVax-S and purified rabies virus antigen from RabiVax-S vaccine were provided by The Serum Institute of India (Pune, India). Anti-rabies glycoprotein monoclonal antibody was obtained from BioRad (Hercules, *CA*).

### Spray-drying

Purified RABV antigen was formulated at a concentration of 5 IU/mL in 9.5 % trehalose, 2.5 % HES, 0.02 mM polysorbate 80, 40 mM ammonium acetate, 10 mM l-histidine, pH 6.5. A Buchi B-290 spray-dryer equipped with a high-performance cyclone separator was used to spray-dry the formulated antigen. Dehumidified air at an inlet temperature of 75 °C and a flowrate of 32 m^3^/hr was used as a drying gas. Nitrogen gas was used in the atomizing nozzle at a flowrate of 414 L/hr, while the liquid sample was fed at 0.5 mL/min. These conditions yielded a spray-drier outlet temperature below 50 °C.

Following spray-drying, powders were removed from the sample collector and aliquoted into 5-mL glass vials. Vials were placed on the shelf of an FTS Systems LyoStar II lyophilizer (Warminster, PA), where they were held overnight at 60 mTorr and 40 °C. Vials were then backfilled with dry nitrogen gas, sealed using DWK butyl rubber stoppers and aluminum caps (Millville, NJ) and stored at 4 °C until use.

### Atomic layer deposition

Using a custom-built fluidized bed atomic layer deposition reactor, spray-dried powders were coated with 150 cycles or 250 layers of alumina using gas-phase injections of trimethylaluminum and water as previously described^17^. Al_2_O_3_ content of the resulting powders was determined by weight after calcining to remove volatilizable excipients.

### Immune response to RABV vaccines

Immunogenicity studies were conducted in BALB/c mice under the University of Colorado Boulder Institutional Animal Care and Use Committee protocol #2318–4DEC2018. BALB/c mice were obtained from Taconic (Hudson, NY). Mice were allowed to acclimate for at least 1 week before use and were 6–9 weeks old at the start of the study. To establish baselines and confirm the absence of pre-existing anti-rabies antibodies, blood samples were collected prior to administration of test vaccines. Ten mice were used in each group and were injected intramuscularly (IM) into the right dorsal thigh with spray-dried or ALD-coated spray-dried powders that were reconstituted or resuspended, respectively, in sterile water for injection. Blood was collected weekly via the submandibular vein. Blood samples were incubated for 24 hrs at 4 °C before processing and centrifuged at 4 °C, 4000 × g for 15 min to pellet cells. Serum was then removed and stored at −70 °C until testing.

### Anti-RABV IgG Titer Determination using Enzyme Linked Immunosorbent Assay (ELISA)

Corning Costar Assay Clear Flat Bottom Medium Binding 96-well assay plates were coated overnight at 4 °C with 50 μL of 0.04 IU/mL RABV antigen. Plates were washed 3 times between steps with a phosphate buffered saline containing 0.05 % polysorbate 20 (PBST). Plates were blocked with 5 % dehydrated skim milk reconstituted in PSBT and incubated at 37 °C for 1 hr. Serum samples added in duplicates were initially diluted 1:100, serially diluted 1:2 across the plate, and incubated for 1 hr at 37 °C. Promega anti mouse-HRP conjugate was added and incubated for 1 hr at 37 °C. ThermoFischer Ultra TMB was added, and the reaction was quenched with 1 M sulfuric acid. Plates were read for optical density (OD) at 450 nm using a BioTek ELx808 plate reader. After subtraction of optical densities measured in serum from pre-immunization bleeds, titers were calculated by plotting OD_450_ *versus* the logarithm of the dilution factor and determining the EC_50_ of the resulting sigmoidal curve using the using the OriginPro^®^ 2022 version 4-parameter sigmoidal fit tool.

### Thermostability of spray-dried and ALD-coated RABV vaccines

Spray-dried and ALD-coated RABV vaccines were incubated for 3 months at 4-, 25-, 40-, and 50 °C prior to intramuscular injection in BALB/C mice. Resulting immune responses were determined in serum samples taken weekly or biweekly for 70 days following the initial injection.

### Karl Fischer moisture determination

Karl Fischer analysis was performed to determine moisture content of spray-dried powders using an ECO KF Titrator from Metrohm (Herisau, Switzerland). Powders were dissolved in formamide and titrated using Methanol and HYDRANAL^™^ Composite 1.

### Differential scanning calorimetry

Glass transition temperatures (T_g_s) of spray-dried microparticles were determined with a TA Discovery DSC 2500 (TA, New Castle, DE) differential scanning calorimeter. Approximately 3 to 5 mg of spray-dried powder were placed in aluminum pans and hermetically sealed. Sample pans were heated from 25 °C to 93 °C, cooled to 25 °C, and reheated to 93 °C at 10 °C/min. T_g_ was determined from the position of the peak maximum in the first derivative of the second heating scan.

### Determination of neutralizing antibody responses

Serum levels of neutralizing antibodies following administration of vaccines to mice were determined by the Kansas State Veterinary Diagnostic Laboratory using a Rapid Fluorescent Foci Inhibition Test^25^ pseudovirus neutralization assay. The assays were conducted on samples of pooled sera from three vaccinated mice; three pooled samples were tested per group.

### Scanning electron microscopy (SEM)

Vaccine powders were adhered to double-sided carbon adhesive tape on an imaging stub, sputter coated with platinum of a thickness of approximately 3 nm using a Cressington 108 Auto/SE Sputter (Liverpool, UK) and imaged with an accelerating voltage of 10 kV using a Hitachi SU3500 Variable Pressure SEM (Hitachi, Tokyo, Japan).

### Statistical analyses

Statistical analyses were performed using Origin Pro 2024 Version (Northhampton, MA). The Shapiro-Wilk Normality Test with a *p*-value of 0.05 was used to test data sets for normality. Statistical differences between groups determined to be normally distributed were determined using one-way ANOVA, with a *p*-value of 0.05 considered as significant. When comparing groups determined to be non-normal, a Kruskal-Wallis ANOVA and Dunn’s post-hoc test were used with a *p*-value of 0.05 considered as significant.

## Results

### Physical characterization of microparticle vaccines

Spray-dried RABV vaccines had moisture contents below 1 %, as determined by Karl Fischer titration. Dry powders were glassy, as evidenced by clear glass transitions during thermal scanning in differential scanning calorimetry (DSC) experiments. T_g_s of spray-dried powders were approximately 80 °C, comparable to T_g_s previously measured for spray dried formulations of viruses with similar excipient compositions^21^.

**Fig. 1.**
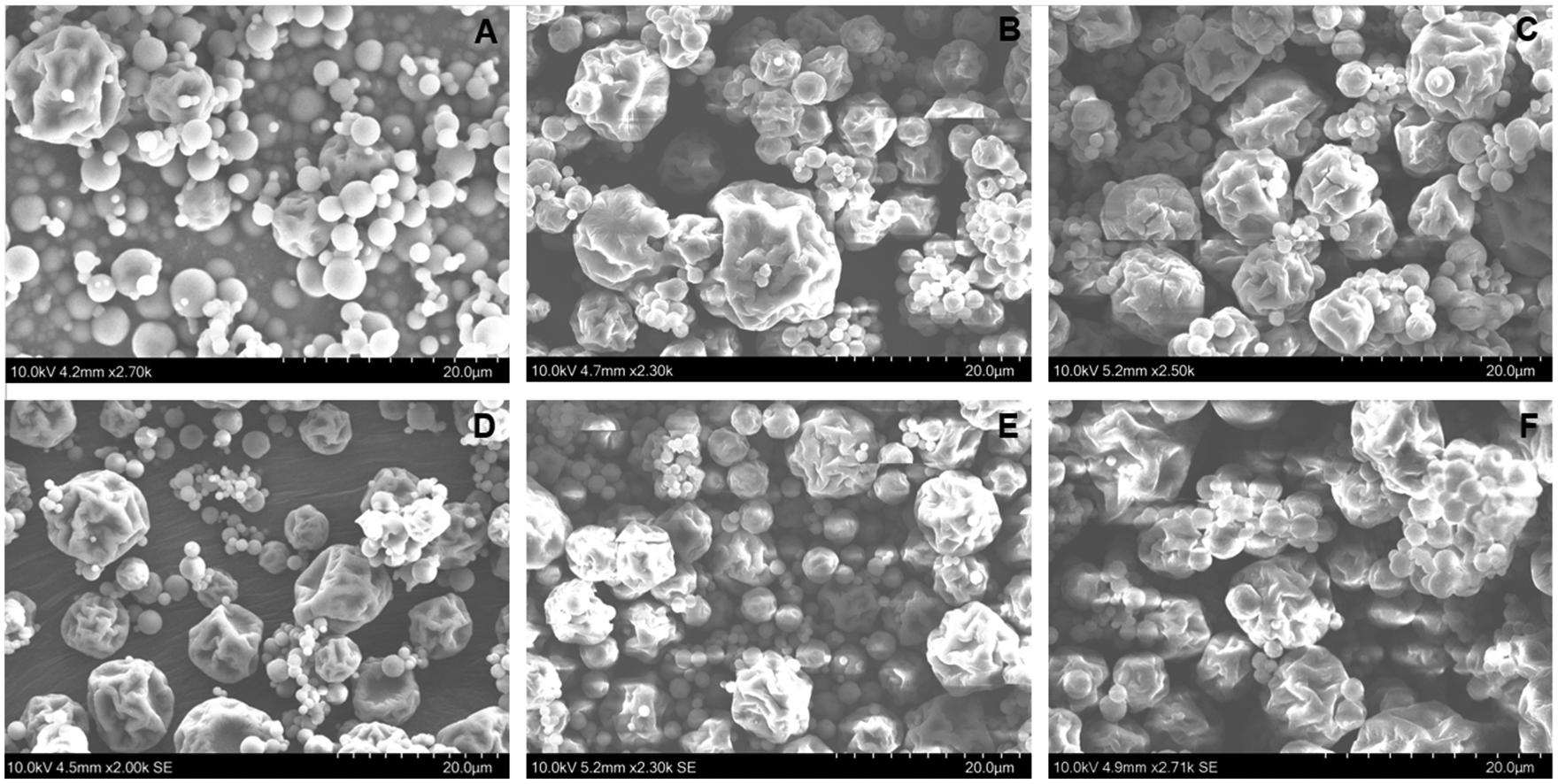
Scanning electron microscopy (SEM) images of RABV vaccine microparticles. **A**) Spray dried RABV vaccine microparticles; **B**) RABV vaccine microparticles after alumina deposition in 150 cycles of ALD; **C**) RABV vaccine microparticles after alumina deposition in 250 cycles of ALD; **D**) Spray dried RABV vaccine microparticles after 3 months incubation at 50 °C; **E**) RABV vaccine microparticles after alumina deposition in 150 cycles of ALD and incubation at 50 °C for 3 months; **F**) RABV vaccine microparticles after alumina deposition in 150 cycles of ALD and incubation at 50 °C for 3 months.

ALD was used to apply either 150 or 250 molecular layers of alumina to the spray-dried powders. Gravimetric analysis following calcination showed alumina contents were 9.8 and 13.9 % in microparticles coated with 150 and 250 ALD-deposited layers of alumina, respectively. Consistent with the expected nanoscopic layer thickness (approximately 35 and 60 nm for 150 and 250 ALD layers, respectively) applied to the micron-sized particles, no discernable changes in particle morphology after ALD coating could be observed by SEM. Further, SEM analyses conducted after incubating the spray-dried and ALD-coated microparticles for 3 months at 50 °C also showed no detectable differences in overall morphology [Fig. 1].

### Thermal stability of spray-dried RABV vaccines

**Fig. 2.**
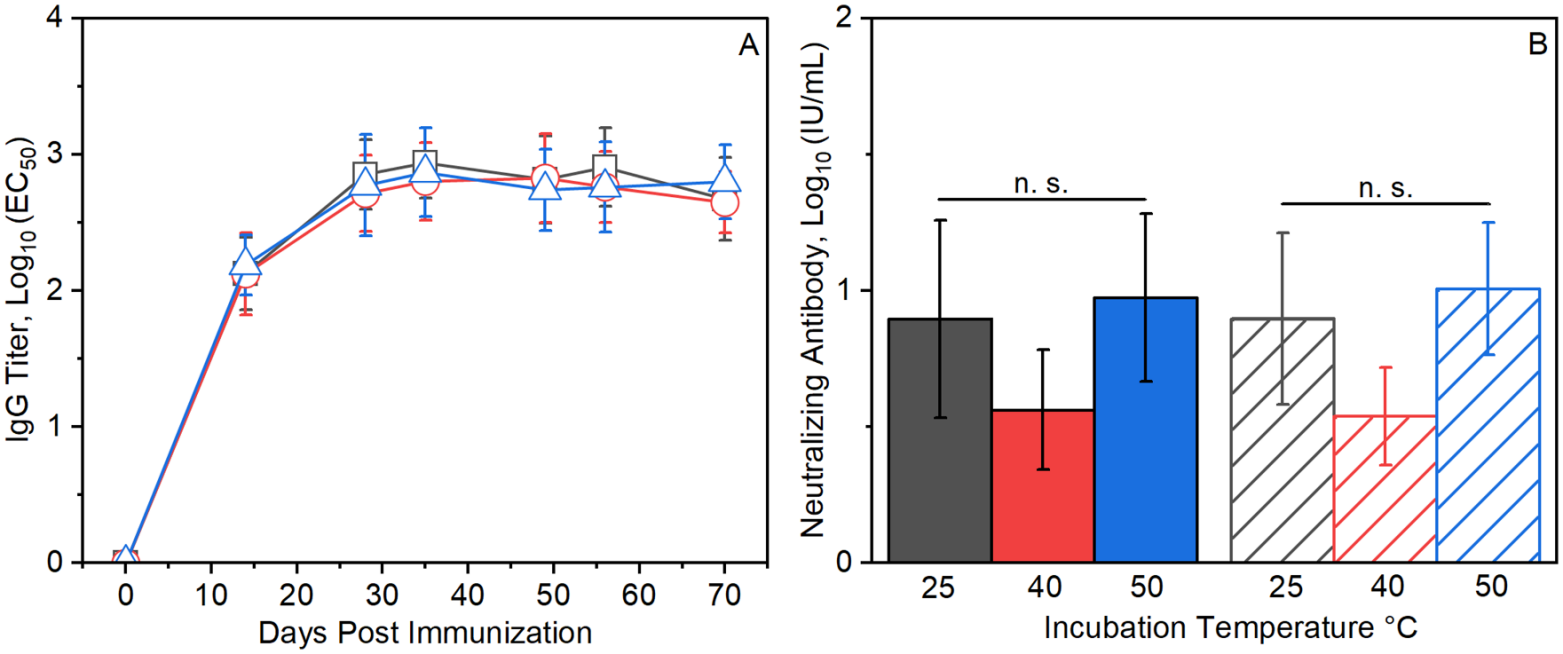
Thermostability of spray-dried RABV vaccines. BALB/c mice were immunized with three 0.04 IU doses of spray-dried and reconstituted RabiVax-S administered on days 0, 7 and 21. **A**) Serum anti-RABV IgG titers *versus* time after initial injection. Spray-dried RABV vaccines were incubated for 3 months at 25 °C (black squares), 40 °C (red circles), and 50 °C (blue triangles) before reconstitution and administration. **B**) Neutralizing antibody titers were measured on days *56* (solid bars) and 70 (hatched bars) following the first injection. For all serum sample timepoints, anti-RABV IgG titers (panel **A**) or neutralizing antibody titers (panel **B**) were indistinguishable for vaccines incubated at the various temperatures (*p* > 0.05).

Spray-dried RABV vaccine powders were incubated for three months at temperatures of 25, 40, and 50 °C prior to reconstitution and administration of 0.04 IU doses to BALB/c mice on days 0, 7 and 21. Immune responses to the vaccines were monitored at weekly or biweekly intervals for 70 days post-injection. Approximately four weeks after the first injection, serum anti-RABV IgG titers reached a plateau of around 10^3^ titer units [Fig. 2A]. Neutralizing antibody titers of approximately 8 IU/mL were measured 56 and 70 days after the first injection [Fig. 2B]. Significantly, even after incubation for three months at 50 °C, the highest temperature tested, no significant changes in anti-RABV IgG responses [Fig. 2A] or neutralizing antibody titers were detected [Fig. 2B].

### Immune response kinetics following injection of ALD-coated RABV vaccines

**Fig. 3.**
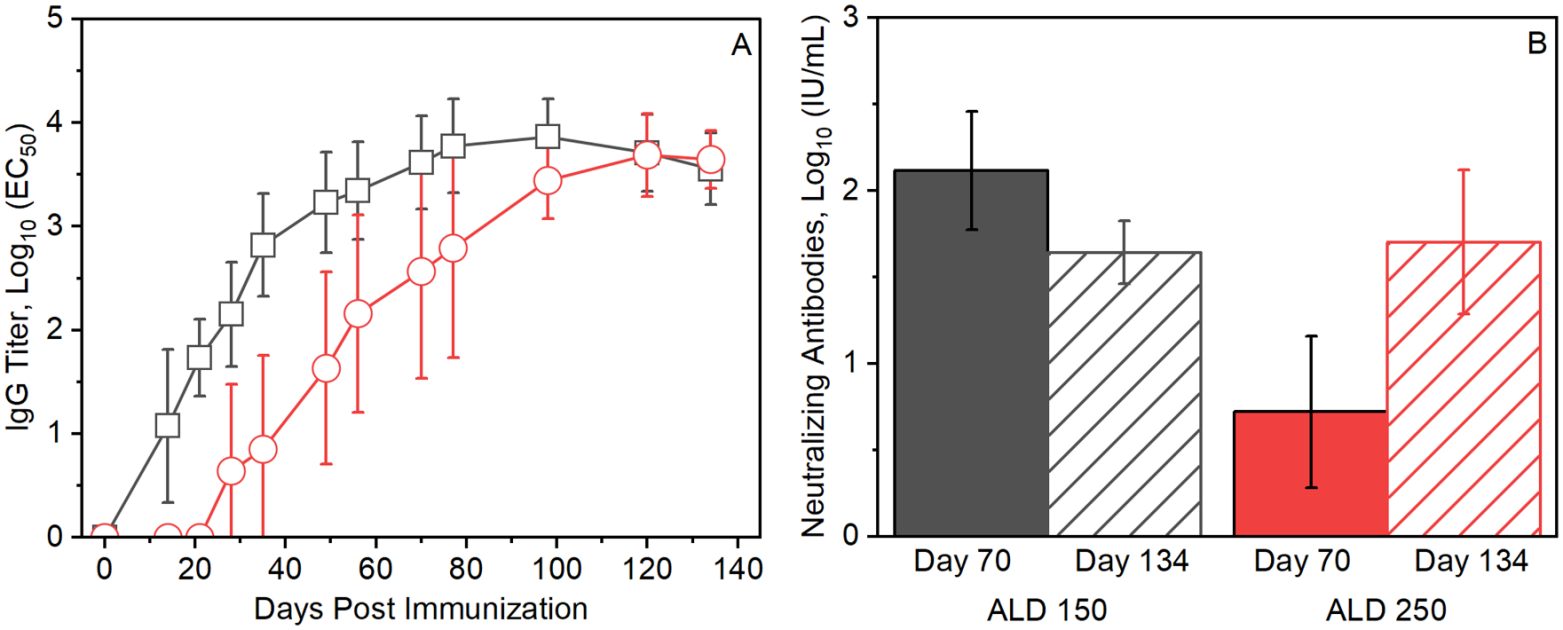
Kinetics of immune response to ALD-coated, spray-dried RABV vaccines. **A**) Serum anti-RABV IgG following a single injection of 0.08 IU Rab V antigen in spray-dried microparticles coated with 150 (black squares) and 250 (red circles) molecular layers of ALD-deposited alumina. **B**) Neutralizing antibody titers measured 70 (solid bars) and 134 days (hatched bars) following injection. Neutralizing antibody titers are averages of measurements of three pooled serum samples, with sera from three mice combined per pool. Colors of the bars correspond to the injection conditions described above in panel.

ALD was used to deposit 150 or 250 molecular layers of alumina on the surfaces of spray-dried RABV vaccine microparticles. Single doses of the microparticles containing 0.08 IU RABV antigen were administered to BALB/c mice, and total anti-RABV IgG antibody and RABV neutralizing antibody titers were measured in serum samples collected over 19 weeks post-injection [Fig. 3].

As expected due to the ALD coating, delayed anti-RABV IgG responses were observed following vaccination with ALD-coated vaccines [Fig. 3A] compared to responses to injections of soluble, reconstituted spray-dried vaccines [Fig. 2A]. Greater delays were observed for vaccines with 250 ALD-applied alumina layers compared to those with 150 ALD layers.

Peak IgG titers following single injections of the ALD-coated microparticle vaccines containing 0.08 IU RABV antigen [Fig. 3A] were 5–10 fold higher than those generated by three injections of reconstituted spray-dried vaccines containing 0.04 IU RABV antigen [Fig. 2A]. Plateau levels of anti-RABV titers measured 12 weeks post injection were indistinguishable (*p* > 0.05) between those achieved with either the 150- or 250-coat vaccines.

Neutralizing antibody levels [Fig. 3B] measured on day 70 post injection of ALD-coated vaccines were higher for microparticulate vaccines with 150 ALD-alumina coats compared to those with 250 coats (*p* < 0.05). However, by day 134 post-injection, neutralizing antibody levels for the two ALD-coated vaccines were indistinguishable (*p* > 0.05). Significantly, neutralizing antibody levels following administration of single 0.08 IU RABV antigen doses of the ALD-coated vaccines [Fig. 3B] were approximately 5–10 times those generated by three doses of uncoated reconstituted spray-dried vaccines (*p* < 0.05) [Fig. 2B].

### Thermostability of ALD-coated RABV vaccine microparticles

**Fig. 4.**
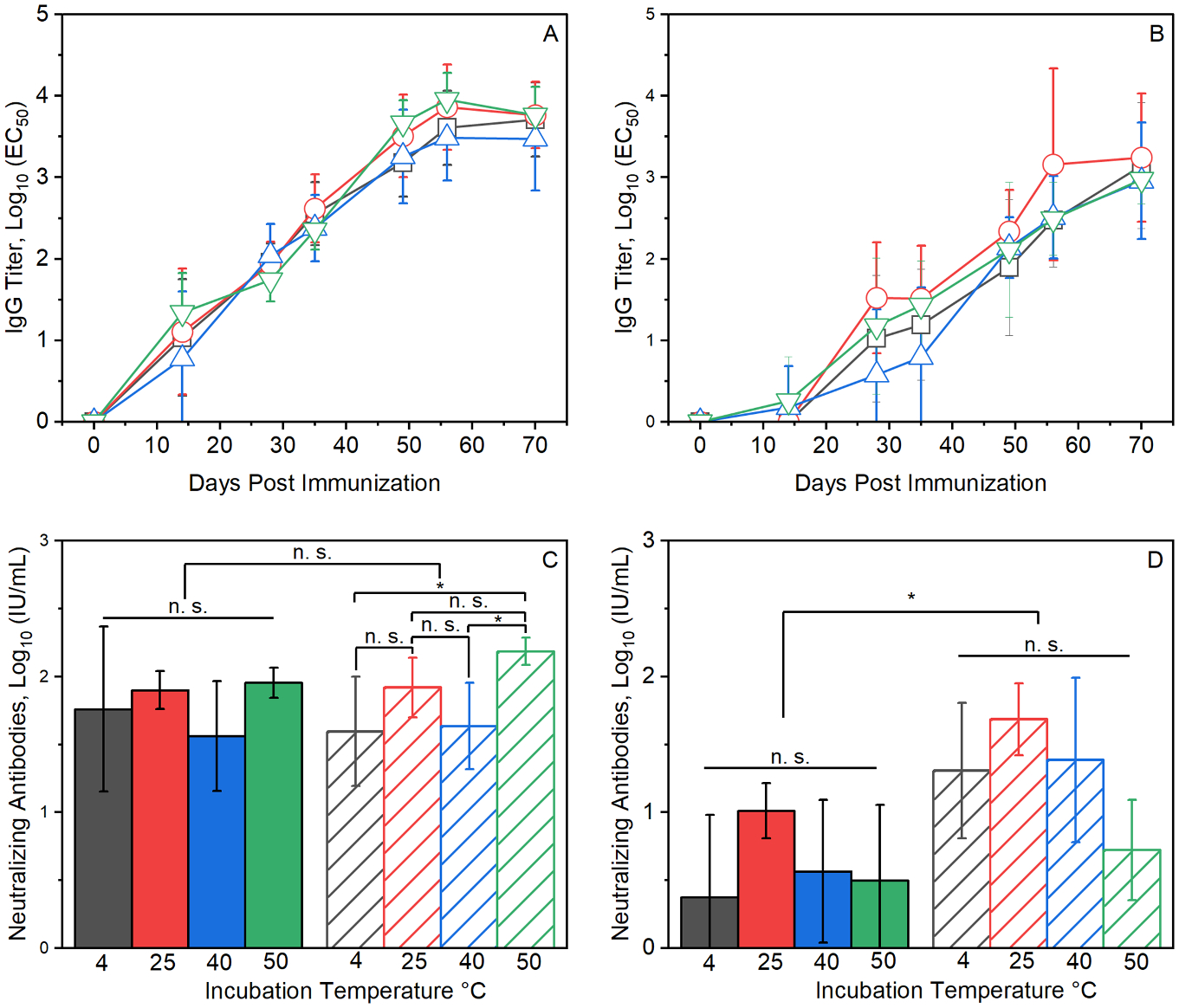
Thermal stability of ALD-coated, spray-dried RABV vaccines. Thermal stability was determined by measuring anti-RABV IgG titers (panels **A** and **B**) and neutralizing antibody titers (panels **C** and **D**) generated in BALB/c mice following administration of single 0.08 IU RABV antigen doses in spray-dried microparticles coated with 150 molecular layers of alumina (panels **A,C**) or 250 molecular layers of alumina (panels **B,D**). Prior to administration, ALD-coated samples were incubated for 3 months at 4 °C (black squares, bars), 25 °C (red circles, bars), 40 °C (blue triangles, bars), and 50 °C (green inverted triangles, bars). Neutralizing antibody titers were measured in sera collected on days 56 (solid bars) and 70 (hatched bars) post-injection.

Spray-dried RABV vaccine microparticles were coated with 150 or 250 molecular layers of alumina by ALD and incubated for three months at 4, 25, 40 or 50 °C before being administered to mice in a single intramuscular injection containing 0.08 IU of RABV antigen. Anti-RABV IgG titers following immunization with vaccines with 150 ALD coats reached levels near 100 and 8000 titer units in sera collected 4- and 10-weeks post injection, respectively [Fig. 4A]. Anti-RABV IgG responses for vaccines with 250 ALD coats showed a delay of approximately two weeks, with titers near 20 titer units detected 4 weeks post-injection [Fig. 4B]. Ten weeks after immunization, anti-RABV IgG titers for vaccines with 250 ALD coats reached 2000 titer units. For both the vaccines with 150 ALD coats and those with 250 coats, no significant differences (*p* > 0.05) in anti-RABV IgG titers were detected as a function of incubation temperature [Figs. 4A, 4B].

Neutralizing antibody titers were measured in serum samples drawn on days 56 and 70 post-injection [Figs. 4C, 4D]. Neutralizing antibody titers for mice immunized with vaccines coated with 150 coats of alumina were independent of vaccine storage temperature and were similar on both days 56 and 70 post-injection. Mice immunized with vaccines coated with 250 ALD coats were similarly storage temperature-independent, but titers were lower in serum samples drawn on day 56 compared to those drawn on day 70.

### Single injection, prime-boost formulations of ALD-coated RABV vaccines

In order to demonstrate how the delayed-release characteristic of ALD-coated RABV vaccines could allow both initial “prime” and delayed-release “boost” doses to be co-administered in a single injection, ALD-coated microparticles containing 0.08 IU RABV antigen were suspended in a liquid formulation containing 0.04 IU of soluble RABV antigen. The resulting “prime-boost” vaccines containing a total 0.12 IU RABV antigen were administered to mice in a single injection. For comparison, one group of mice received three 0.04 IU doses of soluble RABV antigen in a conventional multi-injection dosing schedule on days 0, 7 and 21, while another group received a “prime-only” single dose of 0.04 IU of soluble RABV antigen.

**Fig. 5.**
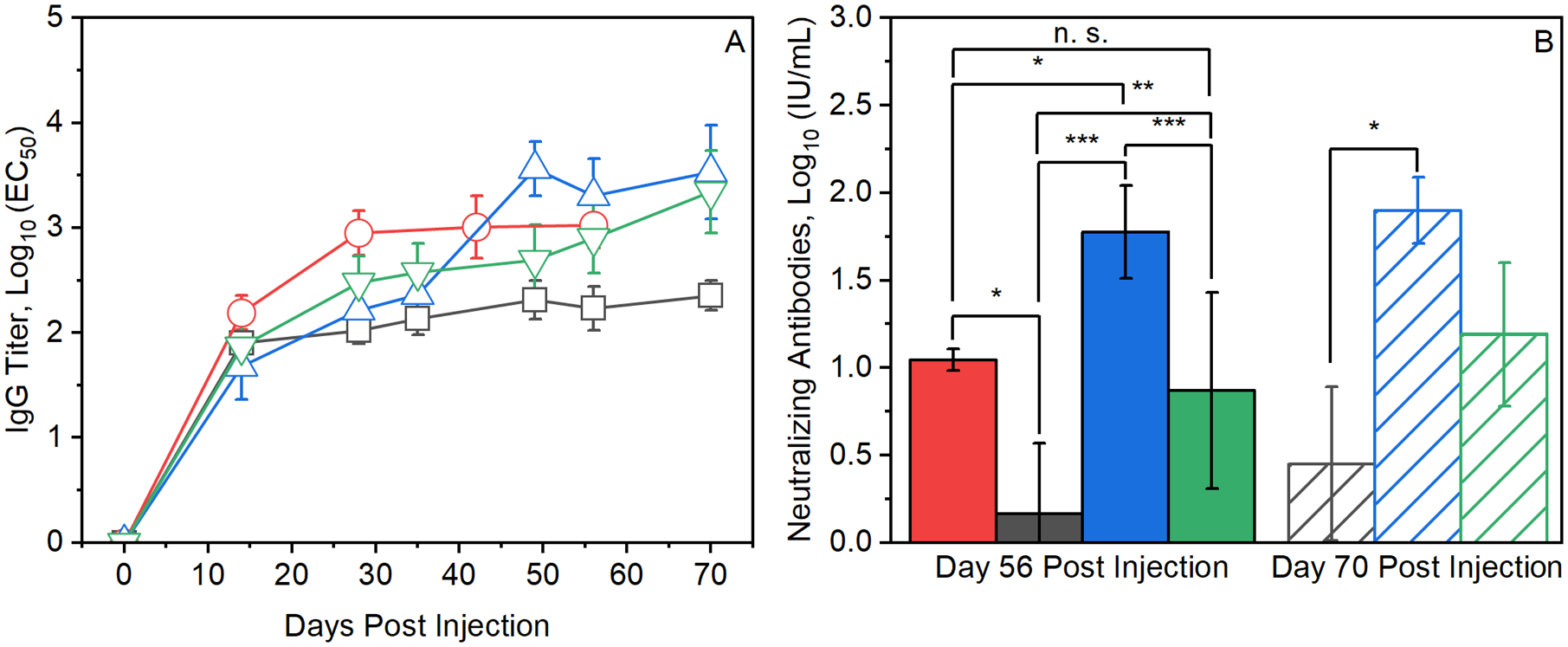
Anti-RABV IgG and neutralizing antibody responses in BALB/c mice following immunization with RABV vaccine. A) Anti-RABV IgG titers in mice immunized with: i) three doses of 0.04 IU RABV administered on days 0,7, and 21 (red circles, *n* = 10); ii) a single priming dose of 0.04 IU RABV (black squares, *n* = 10); iii) a single dose of 0.12 IU RABV formulated as a combination of 0.04 of IU RABV in solution and 0.08 IU spay-dried RABV coated with 150 molecular layers of alumina (green triangles, *n* = 40); and iv) a single dose of 0.12 IU RABV formulated as a combination of 0.04 of IU Rabivax-S in solution and 0.08 IU spray-dried RABV coated with 250 molecular layers of alumina (blue triangles, *n* = 40). B) Neutralizing antibody titers in serum samples collected at 56 and 70 days post-injection. Neutralizing antibody titers were measured in three samples of pooled sera, with sera from three mice combined per pool. Bar colors correspond to the injection conditions described above in panel A.

Mice receiving the “prime-only” dose of 0.04 IU soluble RABV antigen showed a weak anti-RABV IgG response that reached a plateau of approximately 100 titer units by 2 weeks post injection [Fig. 5A] and correspondingly low neutralizing antibody titers measured at both 56 and 70 days post-injection [Fig. 5B]. Mice administered the three-dose series of 0.04 IU injections of soluble RABV antigen developed anti-RABV IgG titers that plateaued at approximately 1000 titer units by day 28 (one week after the last injection), with neutralizing antibody titers near 10 IU.

Two weeks after administration, mice receiving single injections of prime-boost formulations containing RABV vaccine microparticles coated with 150 alumina layers suspended in a soluble priming dose had anti-RABV IgG titers that matched those resulting from the single, prime-only injections [Fig. 5A]. However, in contrast to the plateau in titers seen in the prime-only group, anti-RABV IgG titers in the group receiving single injections of the prime-boost vaccines continued to rise, eventually surpassing even the titers generated by the three-dose series of soluble antigen. Neutralizing antibody titers measured on days 56 and 70 [Fig. 5B] were approximately twice those generated by the three-dose series of soluble RABV antigen.

Single-shot, prime-boost vaccines where the boosting dose was coated with 250 ALD layers yielded anti-RABV IgG titers that initially were similar to those produced by the 150 ALD layer prime-boost formulations. But near the anticipated antigen release time at five weeks post-injection, anti-RABV IgG titers increased sharply, eventually reaching a plateau near 3000 titer units, approximately three times those generated by the three-dose series of soluble Rabivax-S [Fig. 5A]. Neutralizing antibodies measured in sera collected on days 56 and 70 post-injection were approximately twice those measured on day 56 for the three-dose series, although the difference was not significant (*p* > 0.05) [Fig. 5B].

## Discussion

A common strategy for thermostabilizing vaccines is to embed antigens within organic glass matrices formed by lyophilization from solutions containing trehalose, sucrose, or other sugars^26^. For example, even after storage for up to 6 months at 40 °C a lyophilized mRNA-lipid nanoparticle rabies vaccine conferred protection in mice^27^, lyophilized Ebola virus vaccines retained full immunogenicity after 4 months of incubation at 40 °C^28^, and lyophilized human papilloma virus vaccines retained immunogenicity after 12 weeks at 50 °C^29^. Similar stabilization can be achieved when glassy formulations are prepared by spray-drying, *e.g*., as shown by Gomez et al. for spray-dried tuberculosis vaccines^19^. In this study, no losses of anti-RABV IgG or neutralizing antibody titers were detected when spray-dried RABV vaccines were administered after three months of storage at temperatures up to 50 °C [Fig. 2]. Although the low concentration of inactivated virus used in rabies vaccines precludes direct examination of antigen structure, the retention of both anti-RABV IgG and neutralizing antibody titers after processing and storage suggests that conformational structure is retained as well.

In glasses at temperatures below their glass transition temperature T_g_, molecular motions are greatly reduced, resulting in reduced reactivity for embedded antigens. The spray-dried RABV antigen powders had T_g_s above 80 °C. Thus, alumina could be deposited on the surface of spray dried RABV antigen powders in a fluidized bed ALD reactor operated at 70 °C without adversely affecting the immunogenicity of the inactivated rabies virus.

Previously, vaccines against human papilloma virus prepared by spray-drying and ALD with alumina were shown to be thermostable for three months at temperatures up to 70 °C^16^. Consistent with these earlier results, anti-RABV IgG and neutralizing antibody immune responses to spray-dried and ALD-coated RABV antigen microparticle vaccines were unaffected by incubation for three months at temperatures as high as 50 °C. Thus, cold-chain requirements for an ALD-coated RABV antigen vaccine might be relaxed from the current of 2–8 °C storage temperature requirement for Rabivax-S^10^, facilitating logistics for transportation storage and delivery, especially in low- and middle-income countries with limited cold-chain capabilities.

The timing of release of antigens from ALD-coated vaccines and subsequent immune response to the released antigens can be controlled by the number of molecular layers of alumina that are deposited^17,18^. Immune responses to vaccines coated with 250 ALD layers were delayed by approximately two weeks compared to vaccines with 150 ALD layers. [Figs. 2, 3]. In addition to controlled release, ALD-coated RABV vaccines yielded more robust antibody responses than uncoated, soluble preparations. Single doses of ALD-coated vaccines yielded anti-RABV IgG titers and neutralizing antibody titers that were 5–8 fold higher than those resulting from a three-dose series of uncoated, soluble Rabivax-S. [Fig. 4].

Thermostability and controlled release characteristics of ALD-coated vaccines allow the creation of single injection, multidose formulations. Single injections comprising suspensions of ALD-coated RABV vaccine microparticles in solutions of uncoated RABV antigen [Fig. 5] allowed simultaneous administration of a prime dose and a delayed release boost dose in a single injection. The immune responses to these single-injection, prime-boost ALD-coated vaccines were higher than responses to a complete three-dose series of vaccinations with conventional liquid formulations, offering the possibility that the number of doses required for rabies PEP might be reduced if vaccines were administered as prime-boost ALD-coated microparticle formulations.

## Conclusions

Current instability and multi-dose requirements for rabies vaccines provide an obstacle for widespread delivery of the vaccines for pre- and post-exposure RABV prophylaxis. Spray-drying of RABV vaccine antigens from glass-forming polysaccharide solutions confers thermostability to the vaccine microparticles, allowing the vaccines to be stored for three months to temperatures as high as 50 °C without loss of immunogenicity. Further, the application of nanoscopic layers of alumina to the surface of the spray-dried microparticles by atomic layer deposition provides controlled release of antigens, enabling prime and boost injections to be administered in single injections. These ALD coated microparticle RABV vaccines are not only stable for months at elevated temperatures but also generated superior immune responses following single injections compared to those observed following three injections of conventional liquid RAbV antigen formulations. Thus, we anticipate that spray-dried and ALD-coated RABV formulations may help alleviate thermal instability and multi-dose administration requirements that currently pose major challenges to effective, widespread RABV vaccination.

Limitations of the current study relate largely to its proof-of-concept stage. Although no adverse effects or injection site reactions were noted for any of the mice vaccinated in the study, detailed toxicity and dose ranging studies have not been conducted, and materials were produced and tested under non-GMP conditions. Further, kinetics of *in vivo* release of inactivated rabies virions could only be inferred from the resulting immune response, as only very low concentrations of inactivated virions, released over the course of a week or more are required in the current ALD-coated rabies vaccine formulation.
